# Thermodynamic Coupling between Folding Correctors and the First of Dimerized Nucleotide Binding Domains in CFTR

**DOI:** 10.1021/acsbiomedchemau.5c00014

**Published:** 2025-07-30

**Authors:** Guangyu Wang

**Affiliations:** † Department of Physiology and Membrane Biology, 8789 University of California School of Medicine, Davis, California 95616, United States; ‡ Department of Drug Research and Development, Institute of Biophysical Medico-chemistry, Reno, Nevada 89523, United States

**Keywords:** allosteric pathway, cooperative folding, energy landscape, melting threshold, noncovalent thermoring structure, protective folding, protein stability

## Abstract

The most common cystic fibrosis mutation is the F508del mutation in the human cystic fibrosis transmembrane conductance regulator (hCFTR), which causes misfolding of the first of two nucleotide binding domains (NBD1/2), preventing Mg/ATP-dependent NBD dimerization for normal function. Although folding correctors elexacaftor/VX-445 and lumacaftor/VX-809 have been combined to correct the NBD1 misfolding, the exact correction pathway is still unknown. In this study, the constrained tertiary noncovalent interaction networks or the thermoring structures of dimerized NBD1 in hCFTR/E1371Q with or without F508del were analyzed to identify the weakest noncovalent bridge as the final post-translational tertiary folding of dimerized NBD1 in response to folding correctors. These computational analyses suggested that hCFTR primarily used cooperative folding between α- and β-subdomains in dimerized NBD1 as the last step upon binding of the potentiator ivacaftor/VX-770. However, the binding of folding correctors allosterically protected the α-subdomain from misfolding until subsequent core formation. This thermodynamic protective mechanism, unlike the chaperone-based one in cotranslational NBD1 folding, may restore posttranslational NBD1 folding for tight Mg/ATP-mediated NBD dimerization in the F508del mutation and also potentially apply to treating other cystic fibrosis patients with rare mutations.

## Graphical abstract

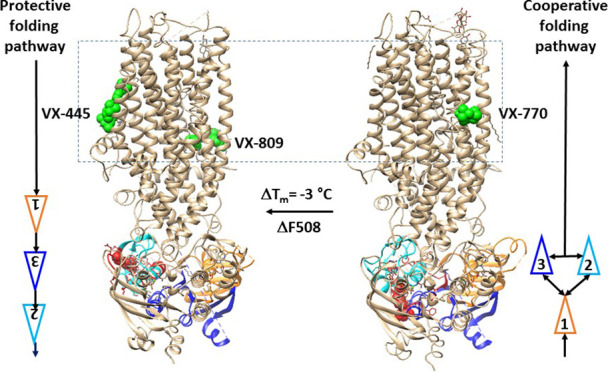

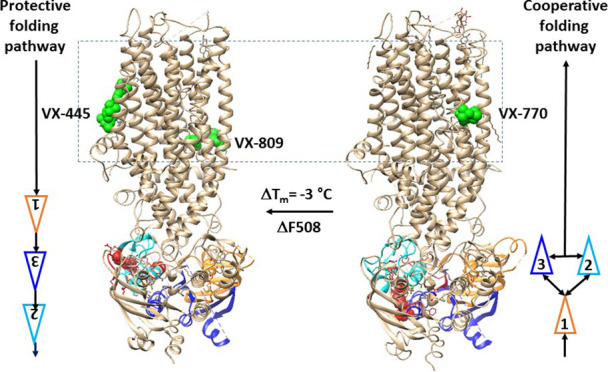

## Introduction

The protecting group strategy is typically used in multistep organic syntheses to control the length and efficiency of the desired reactivity.^1^ Similarly, proteins also employ several protective strategies such as chaperones, quality control mechanisms, and other factors during the folding process to ensure correct 3D structure and to prevent misfolding or aggregation.^2,3^ A good example is the cotranslational folding of the first of two cytosolic nucleotide-binding domains (NBD1/2) from the cystic fibrosis transmembrane conductance regulator (CFTR) among ATP-binding cassette transporters.

The CFTR channel is a multidomain polytopic protein located in the apical membrane of epithelial cells, regulating ion and fluid homeostasis in various tissues.^4^ The NBD1/2 interact with intracellular loops (ICL1-4) extending from two 6-spanning transmembrane domains (TMD2/1) in a domain-swapping manner. A relatively unstructured regulatory (R) domain inserts between NBD1/2 and TMD1/2.^5^ When the R domain is released upon phosphorylation by protein kinase A (PKA), Mg/ATP-mediated NBD1–NBD2 dimerization rearranges the TMD1–TMD2 interactions, leading to channel opening. This opening is stabilized by the hydrolysis-deficient E1371Q mutation.^6–9^

NBD1 consists of an N-terminal (residues 389–491), α-helical (residues 500–564), and parallel-four-stranded β-sheet core (residues 568–603) subdomains.^10^ These subdomains undergo sequential synthesis, starting with ATP-stimulated N-terminal compaction^11^ and ending with C-terminal compaction.^3^ The timing of these folding events is tightly controlled to ensure that α-helical subdomain collapse is delayed until the β-sheet core is synthesized. To prevent misfolding, it is necessary to protect the α-subdomain, possibly with chaperones in the ribosome during synthesis to achieve efficient cotranslational folding without any off-pathway products.^12^

On the other hand, considering that the most common cystic fibrosis mutation F508del damages CFTR folding by destabilizing NBD1 or its interactions with ICL4, leading to a failure of Mg-/ATP-dependent NBD dimerization for normal channel gating,^13–28^ the systematic fluidic grid-like noncovalent interaction mesh networks of NBD1, with or without the interactions with ICL4, have been constrained as thermoring structures with the minimum energy required to stabilize the interactions.^29,30^ Although the biggest thermoring Grid_18_ of the isolated (F508del)­hNBD1 monomer is located in the N-terminal subdomain, and has a calculated melting temperature threshold (*T*_m,th_) of 32 °C to unfold the weakest N396–N445 H-bond,^29^ the biggest thermoring Grid_14_ of the partially dimerized NBD1 of full-length (F508del)­hCFTR with elexacaftor/VX445 bound is positioned in the α-subdomain and has a calculated *T*_m,th_ of 39 °C to unfold the weakest D529–R555 salt bridge. Furthermore, in the tightly dimerized NBD1 of full-length (F508del)­hCFTR with Trikafta (ivacaftor/VX-770, tezacaftor/VX-661 and elexacaftor/VX445) bound, the biggest Grid_10_ is also present in the α-subdomain and has a calculated *T*_m,th_ of 49 °C to unfold the weakest Y517–D537 H-bond.^30^

Since the weakest noncovalent interaction for the final posttranslational folding in NBD1 is located in the region that accumulates numerous suppressor mutations, which improve NBD1 stability, CFTR folding efficiency, and (F508del)­hCFTR processing with folding correctors such as VX-445 or VX-809 synergistically,^22–24,31–35^ it is of special interest to explore the hypothesis that the binding of folding correctors initiates a protective posttranslational pathway to prevent NBD1 misfolding in (F508del)­hCFTR in the absence of chaperones.

To test this hypothesis, the thermoring structures of dimerized NBD1 of human cystic fibrosis transmembrane conductance regulator (hCFTR) with or without F508del were further analyzed in the presence of single or combined modulators. The results demonstrated that the weakest Y517–D537 or D529–R555 H-bond appeared in the α-subdomain of dimerized NBD1 from hCFTR with or without F508del upon the binding of single or combined folding correctors such as VX445, VX-661 or VX-809. In contrast, the least-stable Q525–E585 H-bond linked both α- and β-subdomains in hCFTR with the potentiator ivacaftor/VX-770 bound. Therefore, dimerized NBD1 in full-length hCFTR employed a cooperative pathway in the final posttranslational folding. However, the binding of the folding corrector to hCFTR stimulated a protective pathway in dimerized NBD1, which was used to correct for the structural defect of (F508del)­hCFTR.

## Computational Methods

### Data Mining Resources

Four cryo-EM structures of phosphorylated and Mg/ATP-bound hCFTR/E1371Q constructs in an activated state at 4 °C were selected for thermoring analysis. Three structures with F508 included 6O2P with VX-770 bound (model resolution = 3.3 Å),^36^ 7SV7 with VX-661 bound (model resolution = 3.8 Å),^28^ and 7SVD with VX-809 bound (model resolution = 2.7 Å).^28^ One structure without F508 was 8EIO with VX445/VX-809 bound (model resolution = 2.8 Å).^28^

### Standard Methods for Filtering Tertiary Noncovalent Interactions

The standard methods for filtering tertiary noncovalent interactions, along with exact calculations, were the same as those previously used, ensuring accurate and repeatable results.^29,30,37–43^ UCSF Chimera was used to review potential stereoselective or regioselective intradomain lateral tertiary noncovalent interactions along the single polypeptide chain of hNBD1 with or without F508. These interactions included salt bridges, H-bonds, and lone pair/CH/cation–π interactions between paired amino acid side chains. When a backbone NH or CO at a residue position was within a cutoff distance for an H-bond with a side chain of a nearby residue, that H-bond was also considered. Detailed cutoff distances and interaction angles can be obtained in the online Supporting Information (Tables S1–S4). Notably, momentary fluctuation-induced perturbations in tertiary noncovalent interactions during protein dynamics were not taken into account. In this study, approximately 36–43 different tertiary noncovalent interactions were identified along the single polypeptide chain from L383/E384 to L636/Q637 of NBD1 on each protomer.

### Mapping the Energy Landscape of Tertiary Folding of NBD1 Using the Grid Thermodynamic Model

The same grid thermodynamic model that was previously validated was used to map the energy landscape of tertiary folding of NBD1 in full-length hCFTR with or without F508.^29,30,37–43^ Briefly, a black line represented the specific polypeptide chain from the N-terminal to the C-terminal of NBD1 while colorful lines represented specific noncovalent interactions linked by side chains of paired protein residues (colorful arrows). When these lines formed a systematic fluidic grid-like mesh network, each noncovalent interaction had at least two paths between paired residues. A direct path was the interaction itself, while a reverse path consisted of the nearby peptide segment and other noncovalent interactions. Since the reverse path could be shortened by Floyd-Warshall’s Algorithm as the minimal free or silent residues that did not involve any noncovalent interactions,^44^ the shortest direct and reverse pathways created a thermosensitive ring or thermoring, denoted as Grids, where “s” represented the size of a thermoring or the total number of free or silent residues to control the weakest noncovalent interaction within it. For example, when E391 formed an H-bond with K447 in Figure 2a, if a direct path started from E391 and ended at K447, then the peptide segment from K447 to E391 paved a reverse path. Since another adjacent reverse path from K447 to F446, M394, and E391 had a length of 2 (the total number of free amino acids in peptide segments K447 to F446 and 394 to 391), and no other possible adjacent reverse paths were shorter than 2, the shortest round path from E391 to M394, F446, and K447 and back to E391 lined a thermoring Grid_2_ with a two-residue size to control the least-stable E391–K447 H-bond.

Once each tertiary noncovalent interaction was assigned a unique size, the largest thermoring could be identified to trace the weakest noncovalent interaction along the single peptide chain. Meanwhile, the total noncovalent interactions and grid sizes along the same polypeptide chain could be summed up and displayed by black and cyan circles next to the mesh network map.

### Calculation of the Melting Temperature Threshold (*T*_m,th_) of NBD1

The same empirical equation and coefficients used in previous studies on temperature-dependent structures were applied to calculate the melting temperature threshold (*T*_m,th_) of NBD1 ^29,30,37–43^

Tm,th(C°)=34+(n−2)×10+(20−s)×2 1

where *n* represents the total number of basic H-bonds (each approximately 1 kcal/mol) that are calculated to be approximately equal in stability to the least-stable or weakest noncovalent interaction controlled by the biggest grid^45^ and s is the grid size used to control the least-stable noncovalent interaction in the biggest grid.

### Evaluation of the Grid-Based Systemic Thermal Instability (*T*_i_) of NBD1

The same empirical equation used in previous studies on temperature-dependent structures was utilized to calculate the systematic thermal instability (*T*_i_) of NBD1 ^29,30,37–43^

2 $$T_{i}=S/N$$

where *S* and *N* are the total grid sizes and the total noncovalent interactions along a specific polypeptide chain of NBD1, respectively. This calculation allows for evaluation of NBD1’s compact conformational entropy or flexibility.

## Results

### Biggest Thermoring of Dimerized (F508del)­hNBD1 Is Located in the α-subdomain upon VX-445/VX-809 Binding to TMD1/2

The specific binding of VX-809 and VX-445 to TMD1 and the TMD2/lasso interface is sufficient to restore normal Mg/ATP-mediated NBD1–NBD2 dimerization of (F508del)­hCFTR/E1371Q without the potentiator VX-770.^28^ Therefore, it is intriguing to explore the impacts of folding correctors on the location and stability of the biggest thermoring in (F508del)­hNBD1.

The dimerized (F508del)­hNBD1 (PDB, 8EIO) is a single polypeptide spanning from L383 to L636 with a disordered regulatory insertion (RI) (residues E403 to L436).^28^ Along this peptide chain, forty-four intradomain noncovalent interactions via amino acid side chains were located in the N-terminal, α- and β-core subdomains (Figure 1a). Along with tight Mg^2+^ binding to T465, Q493, and D572 and tight ATP binding to W401, K464, T465, S466 and Q493, the Q552–D529–R555 bridges formed a smaller Grid_2_ in the α-subdomain while F575, F587, and H609 produced the smallest Grid_0_ in the β-subdomain (Figure 1a). Collectively, a ratio of a total of 69 grid sizes to a total of 44 noncovalent interactions was calculated as a systematic thermal stability (*T*_i_) of 1.57 (Table 1). Therefore, dimerized hNBD1 was tightly folded. Meanwhile, hNBD1 also interacted with ICL4 via E474–R1066 and E543–T1057 H-bonds and several π interactions, such as Y380–R1066, E474–W1063, and W496–F1074 (Figure 1b).

**Figure 1:**
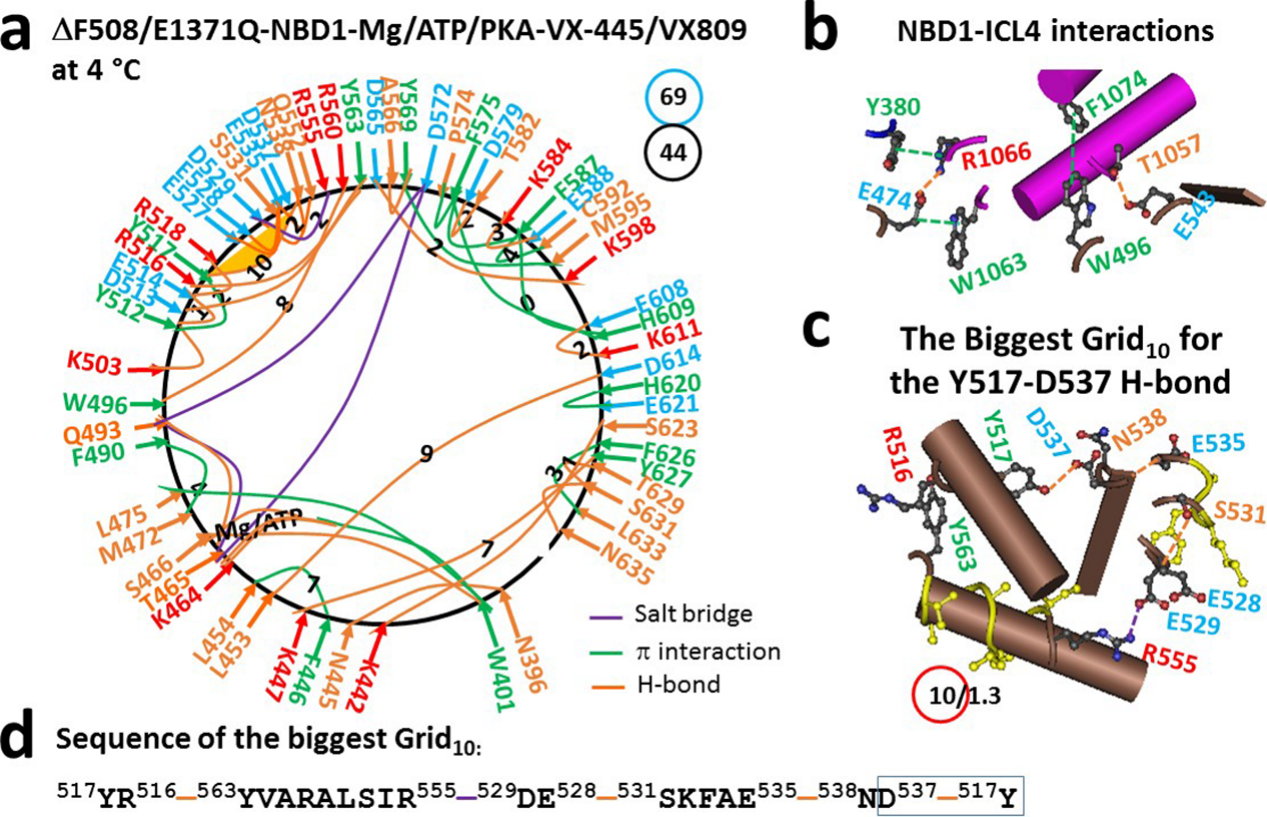
Thermoring structures of phosphorylated hCFTR/E1371Q/ΔF508 with VX-445 and VX-809 bound in the activated state at 4 °C. (a) The grid-like noncovalently interacting mesh network based on the cryo-EM structure of hCFTR/E1371Q/ΔF508 with VX-445 and VX-809 bound in the presence of Mg/ATP/PKA at 4 °C (PDB ID, 8EIO, 2.8 Å). Salt bridges, H-bonds, and π interactions are colored purple, orange, and green, respectively. The constrained grid sizes required to control the least-stable noncovalent interactions in the grids are labeled with black numbers. The least-stable Y517–D537 H-bond in the biggest Grid_10_ is highlighted. The total grid sizes and the total grid size-controlled noncovalent interactions along the single peptide chain of NBD1 from L383 to L636 are shown in cyan and black circles, respectively. (b) Noncovalent interactions at the NBD1/ICL4 interface (Y380 is at the TMD1–NBD1 linker). (c) The structure of the biggest Grid_10_ with a 10-residue size to control the least-stable Y517–D537 H-bond. The grid size and the equivalent basic H-bonds for the least-stable noncovalent interaction are shown in and near a red circle. (d) The sequence of the biggest Grid_10_ to control the least-stable Y517–D537 H-bond in the blue box.

**Table 1:** Grid Thermodynamic Model-Based New Parameters of NBD1 in hCFTR Constructs

construct	hCFTR/E1371Q			
PDB ID	6O2P	7SV7	7SVD	8EIO
F508	+	–	–	–
Mg/ATP (10 mM)/PKA	+	+	+	+
bound modulator, VX-	770	661	809	445/809
sampling temperature, °C	4	4	4	4
NBD dimerized	+	+	+	+
normal Mg^2+^ site	+	–	–	+
name of the biggest grid in α-subdomain	grid_8_	grid_9_	grid_12_	grid_10_
involving α/β-subdomain	+	–	–	–
grid size (s)	8	9	12	10
# of energetically equivalent basic H-bonds (n) controlled by grid_s_	1.2	2.0	2.0	1.3
total noncovalent interactions (N)	46	44	38	44
total grid sizes (S), a.a	63	66	73	69
systematic thermal instability (*T*_i_)	1.37	1.50	1.92	1.57
calculated *T*_m,th,_ °C	50	56	50	47

On the other hand, despite the tight coupling between the N- and C-termini via K442–S623, Y627–K447 and L453–D614 backbone H-bonds, the biggest Grid_10_ was found in the α-subdomain along with weakened interfacial interactions between NBD1 and ICL4. With a 10-residue size controlling the least-stable Y517–D537 H-bond, which was equivalent to 1.3 basic H-bonds, the calculated melting threshold (*T*_m,th_) for the thermoring from Y517, R516, Y563, R555, D529, E528, S531, E535, N538, D537, and back to Y517 was about 47 °C (Table 1), higher than the *T*_m,th_ of 39 °C for (F508del)­hNBD1 with VX-445 bound.^30^ This result was consistent with the notion that the corrector VX-445 fails to confer enough NBD1 stability to poorly responsive variants.^32^

If the location of the biggest Grid_10_ in the α-subdomain is a result of weakened ICL4–NBD1 interactions in (F508del)­hCFTR rather than the binding of VX445 and VX-809 to TMD1/2, then the binding of folding correctors to hCFTR/E1371Q should reposition the biggest thermoring in NBD1. To investigate this, the thermoring structures of NBD1 in hCFTR/E1371Q were analyzed in response to different modulators.

### Biggest Thermoring of Dimerized hNBD1 Involves Both α- and β-Subdomains upon VX-770 Binding to the TMD1/TMD2 Interface

After the potentiator VX-770 bound to the TMD1/TMD2 interface of hCFTR/E1371Q, forty-six intradomain noncovalent interactions via amino acid side chains were identified in dimerized NBD1 (Figure 2a). NBD1 is a single polypeptide from L383 to Q637 and includes the disordered RI from E410 to L436.^36^ When compared with (F508del)­hCFTR/E1371Q with VX-445 and VX809 bound (PDB, 8EIO), although the ICL4–NBD1 interactions were enhanced by the additional M469–W1063 and F1068/Y1073–F508–F1074–L1065 π interactions (Figure 2b), ATP still tightly bound to W401, G463, K464, T465, S466, and Q493, along with the additional E391–K447 and T398–L441 H-bonds and the supplemental M394–F446 and F400–F409 π interactions (Figure 2a). As a result, several changes were observed in the α- and β-subdomains (Figure 2a). For example, when the D529–Q552 H-bond was replaced with the R516–Y563–Y517 π interactions in the α-subdomain, the Y569–M595 π interaction as well as the Y565–K598, K584–E588, E608–K611 and S631–N635 H-bonds were disrupted in the β-subdomain. In this case, when the K503–Y512–Y517–D537–K503 noncovalent bridges formed the new smallest Grid_0_ in the α-subdomain, Q525 H-bonded with E585 via their side chains, coupling both α- and β-subdomains. Meanwhile, the T465–D572, A462–G622, and K447–Y627 H-bonds still linked the N- and C-termini together (Figure 2a). However, the total noncovalent interactions and grid sizes changed from 44 to 46 and from 69 to 63, respectively. Therefore, the systematic thermal instability (*T*_i_) decreased from 1.57 to 1.37.

**Figure 2:**
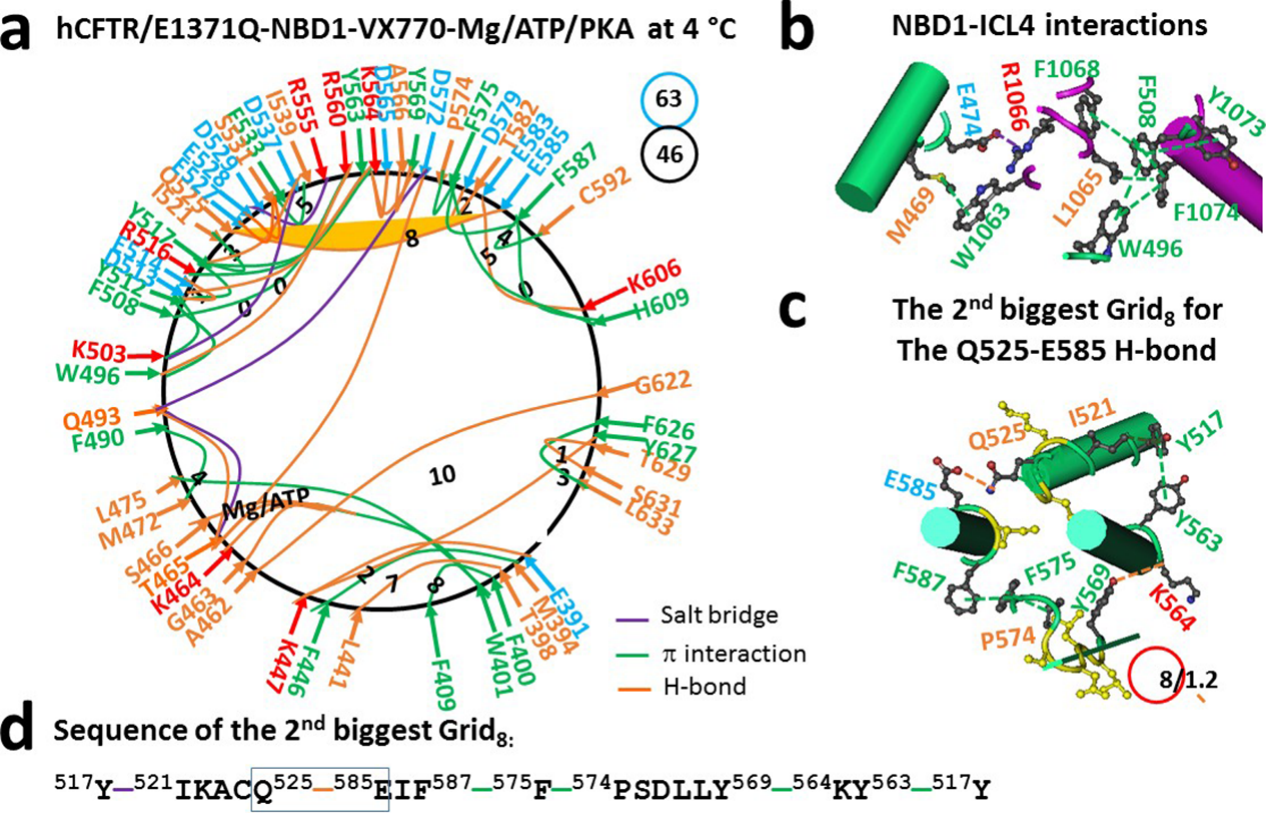
Thermoring structures of phosphorylated hCFTR/E1371Q with VX-770 bound in the activated state at 4 °C. (a) The grid-like noncovalently interacting mesh network based on the cryo-EM structure of hCFTR/E1371Q in the presence of Mg/ATP/PKA and VX-770 at 4 °C (PDB ID, 6O2P, 3.3 Å). Salt bridges, H-bonds, and π interactions are colored purple, orange, and green, respectively. The constrained grid sizes required to control the least-stable noncovalent interactions in the grids are labeled with black numbers. The least-stable Q525–E585 H-bond in the second biggest Grid_8_ is highlighted. The total grid sizes and the total grid size-controlled noncovalent interactions along the single peptide chain of NBD1 from E384 to Q637 are shown in cyan and black circles, respectively. (b) Noncovalent interactions at the NBD1/ICL4 interface. (c) The structure of the second biggest Grid_8_ with an 8-residue size to control the least-stable Q525–E585 H-bond. The grid size and the equivalent basic H-bonds for the least-stable noncovalent interaction are shown in and near a red circle. (d) The sequence of the second biggest Grid_8_ to control the least-stable Q525–E585 H-bond in the blue boxes.

Notably, along with the additional H-bond between the side chain of Y569 and the backbone CO of K564, the Q525–E585 H-bond in the second biggest Grid_8_ still linked both α- and β-subdomains via a thermoring from Y517 to I521, Q525, E585, F587, F575, P574, Y569, K564, Y563, and back to Y517 (Figure 2c,d). When this H-bond were energetically equivalent to 1.2 basic H-bonds (1.2 kcal/mol), the calculated *T*_m,th_ was about 50 °C (Table 1), which was the same as the *T*_m,th_ of 50 °C in NBD1 of hCFTR/E1371Q (PDB, 6MSM).^30^ Therefore, although ivacaftor/VX-770 destabilizes full-length F508del–CFTR and accelerates channel deactivation at 37 °C,^46^ it had no effect on cooperative folding between the α- and β-subdomains of hCFTR/E1371Q.

### Biggest Thermoring of Dimerized hNBD1 Appears in the α-Subdomain upon VX-661 Binding to TMD1

When VX-770 was replaced with VX-661, the different binding site in TMD1 disrupted the F508–Y1073 and L1065–F1074 π interactions at the NBD1–ICL4 interface, inducing a global change in NBD1 despite intact ATP binding (Figure 3a,b).^28,36^ In the N-terminal subdomain, the M394–F446 and F400–F409 π interactions and the T398–L441 H-bond were substituted with the N396–D443 H-bond. In the α subdomain, the K503–D537 salt bridge as well as the R516/Y517–Y563 π interactions were disrupted. Meanwhile, the K522–E527 salt bridge, the T501–E504 H-bond, and the Y515–S519 π interaction were created. Following the disruption of the Q525–E585 H-bond between α and β subdomains, the E583–K606 and T629–S631 H-bonds, together with the F626–L633 π interaction, were replaced with a new D567–T599 H-bond and the E588–K612 salt bridge. Taken as a whole, the total number of tertiary noncovalent interactions and grid sizes changed from 46 to 44 and from 63 to 66, respectively. Therefore, the systematic thermal instability (T_i_) also increased from 1.37 to 1.50 (Table 1). When the T465–D572, A462–G622, K447–Y627, and D443–S624 H-bonds still coupled the N-terminal with the β-core subdomain, the biggest Grid_9_ was identified in the α subdomain to control the D529–R555 H-bond via a thermoring from D529, E528, S531, F533, I539, D537, Y517, Y512, F508, R560, and R555, and back to D529. When the controlled H-bond was energetically equivalent to 2.0 basic H-bonds, the calculated *T*_m,th_ was about 56 °C (Table 1), which was higher than the *T*_m,th_ of 50 °C of NBD1 in hCFTR/E1371Q (PDB, 6MSM).^30^ Therefore, binding of VX-661 to TMD1 relocated the final folding in the α-subdomain and enhanced the stability of NBD1, regardless of the change in the ICL4–NBD1 interactions.

**Figure 3:**
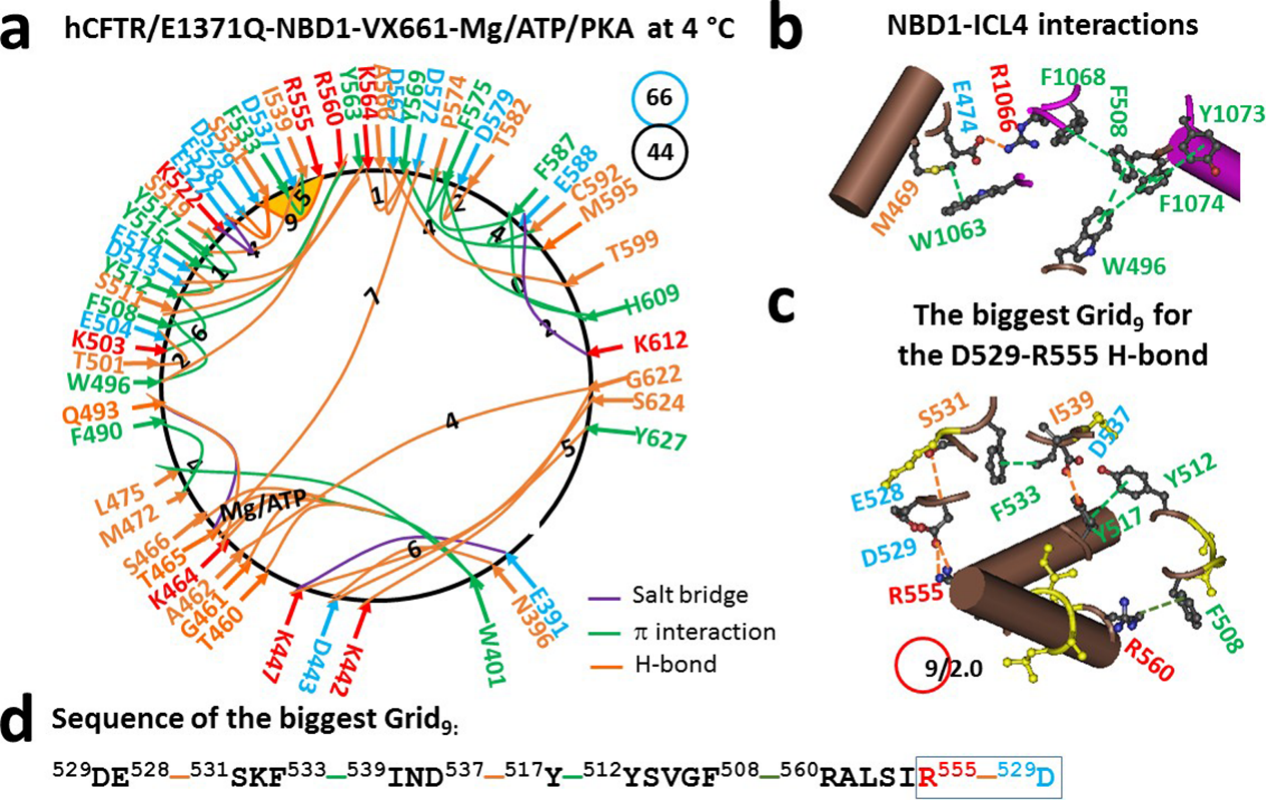
Thermoring structures of phosphorylated hCFTR/E1371Q with VX-661 bound in the activated state at 4 °C. (a) The grid-like noncovalently interacting mesh network based on the cryo-EM structure of hCFTR/E1371Q in the presence of Mg/ATP/PKA and VX-661 at 4 °C (PDB ID, 7SV7, 3.8 Å). Salt bridges, H-bonds, and π interactions are colored purple, orange, and green, respectively. The constrained grid sizes required to control the least-stable noncovalent interactions in the grids are labeled with black numbers. The least-stable D529–R555 H-bond in the biggest Grid_9_ is highlighted. The total grid sizes and the total grid size-controlled noncovalent interactions along the single peptide chain of NBD1 from E384 to Q637 are shown in cyan and black circles, respectively. (b) Noncovalent interactions at the NBD1/ICL4 interface. (c) The structure of the biggest Grid_9_ with a 9-residue size to control the least-stable D529–R555 H-bond. The grid size and the equivalent basic H-bonds for the least-stable noncovalent interaction are shown in and near a red circle. (d) The sequence of the biggest Grid_9_ to control the least-stable D529–R555 H-bond in the blue box.

### Biggest Thermoring of Dimerized NBD1 Also Remains in the α-Subdomain upon VX-809 Binding to TMD1

VX809 and VX661 share the same binding site,^28^ leading to similar effects on the tertiary structure of NBD1 and the NBD1–ICL4 interface (Figure 4a,b). Restoring the L1065–F1074 π interaction at the interface disrupted the weakest Q525–E585 H-bond between the α- and β-subdomains. As a result, in the α-subdomain, the K522–E527 salt bridge, the Y517–D537 H-bond, and the Y515–S519 and F533–I539 π interactions were replaced by the S549–Q552 H-bond and the Y517–Y563 π interaction. Meanwhile, in the β-core subdomain, an H-bond shifted from D567–T599 to E583–H609 along with replacing the E588–K612 salt bridge with the E608–K611 H-bond. Lastly, while the H-bonds shifted from A462–G622 and D443–S624 to L453–D614 and K442–S623, respectively, at the interface between the N- and C-termini, the E391–K447 salt bridge and the N396–D443 H-bond in the N-terminal disappeared, but the F626–L633 π interaction appeared in the C-terminal. Overall, the total grid sizes changed from 66 to 73 along with a decrease in the total noncovalent interactions from 44 to 38 (Figure 4a), resulting in an increase in systematic thermal instability (*T*_i_) from 1.50 to 1.92 (Table 1).

**Figure 4:**
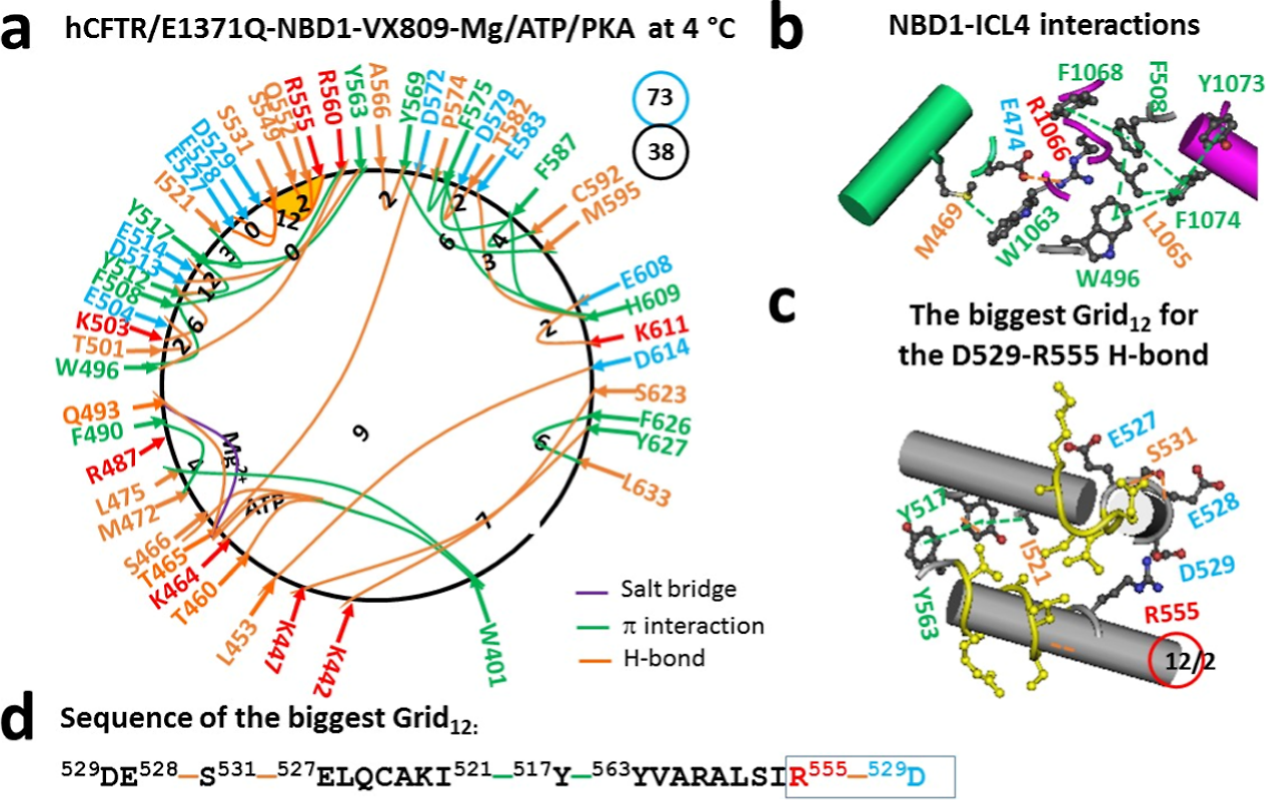
Thermoring structures of phosphorylated hCFTR/E1371Q with VX-809 bound in the activated state at 4 °C. (a) The grid-like noncovalently interacting mesh network based on the cryo-EM structure of hCFTR/E1371Q in the presence of Mg/ATP/PKA and VX-809 at 4 °C (PDB ID, 7SVD, 2.7 Å). Salt bridges, H-bonds, and π interactions are colored purple, orange, and green, respectively. The constrained grid sizes required to control the least-stable noncovalent interactions in the grids are labeled with black numbers. The least-stable D529–R555 H-bond in the biggest Grid_12_ is highlighted. The total grid sizes and the total grid size-controlled noncovalent interactions along the single peptide chain of NBD1 from E384 to Q637 are shown in cyan and black circles, respectively. (b) Noncovalent interactions at the NBD1/ICL4 interface. (c) The structure of the biggest Grid_12_ with a 12-residue size to control the least-stable D529–R555 H-bond. The grid size and the equivalent basic H-bonds for the least-stable noncovalent interaction are shown in and near a red circle. (d) The sequence of the biggest Grid_12_ to control the least-stable D529–R555 H-bond in the blue box.

On the other hand, the weakest D529–R555 H-bond was influenced by the biggest Grid_12_ through a thermoring from D529, E528, S531, E527, I521, Y517, Y563, R555 and back to D529 in the α-subdomain. Hence, the calculated *T*_m,th_ was about 50 °C, which was the same as the *T*_m,th_ of 50 °C for NBD1 in hCFTR/E1371Q (PDB, 6MSM).^30^ In this context, the binding of VX-809 to TMD1 caused a relocation of the final folding in the α-subdomain regardless of the change in the NBD1–ICL4 interactions.

## Discussion

The folding kinetics of NBD1 in hCFTR is complex and can be influenced by various environmental factors. The NBD1-NBD2 dimerization through bound Mg/ATP is essential for normal CFTR activity, but this process, when disrupted by the misfolding of (F508del)­hNBD1, can be restored by the binding of folding correctors such as VX-445 and VX-809 to TMD1/2, highlighting the importance of understanding the final step of NBD1 folding for the development of an allosteric correction pathway. In this study, the effects of different modulators on the thermoring structures of dimerized NBD1 with Mg/ATP bound in full-length hCFTR/E1371Q with or without F508 deletion were examined. The comparative subdomain interactions, *T*_m,th_ values, and drug behaviors suggest that the final step of posttranslational NBD1 folding under physiological condition may involve either a cooperative or protective mechanism, which is lacking in F508-deleted hCFTR. While the cooperative interaction between α and β-subdomains in dimerized NBD1 was still present with the binding of VX-770 to the TMD1/TMD2 interface, the protective step was initiated by the binding of correctors, regardless of the presence of the F508del mutation. Therefore, this protective folding pathway in dimerized NBD1, activated by correctors to rectify the misfolding caused by the F508del mutation, could serve as a potential strategy for developing drugs to treat patients with rare CF mutations in the future.

### The Q525–E585 H-Bond Promotes Cooperative Folding Coupling between α- and β-Subdomains

Several lines of evidence have demonstrated that the coupling between ICL4 and NBD1 facilitates NBD1 and full-length CFTR folding.^23,24,35^ A recent study further showed that the Q525–E585/S589 H-bond between α- and β-subdomains is present in the isolated hNBD1 monomer but absent upon F508 deletion. When the RI (residues 400–439) and the regulatory extension (RE) (residue 645–675) are removed for Mg/ATP-mediated NBD dimerization, it disappears in hNBD1 but appears in (F508del)­hNBD1. It was also found in the (F508del)­hNBD1 monomer with 3S mutations.^29^ In contrast, this H-bond appears again in dimerized NBD1 of full-length hCFTR/E1371Q with or without VX-770 bound but disappears in dimerized NBD1 of full-length hCFTR/E1371Q or (F508del)­hCFTR/E1371Q with VX-661 or VX-809 or VX-445 bound (Figures 1a, 2a, 3a, 4a).^30^ Therefore, although the removal of RI and RE or the introduction of the 3S mutations restores the cooperative folding pathway used in isolated (F508del)­NBD1 from full-length hCFTR, it was compromised in NBD1 of (F508del)­hCFTR with folding correctors bound. In this case, an alternative protective folding pathway was needed (Figure 5).

**Figure 5:**
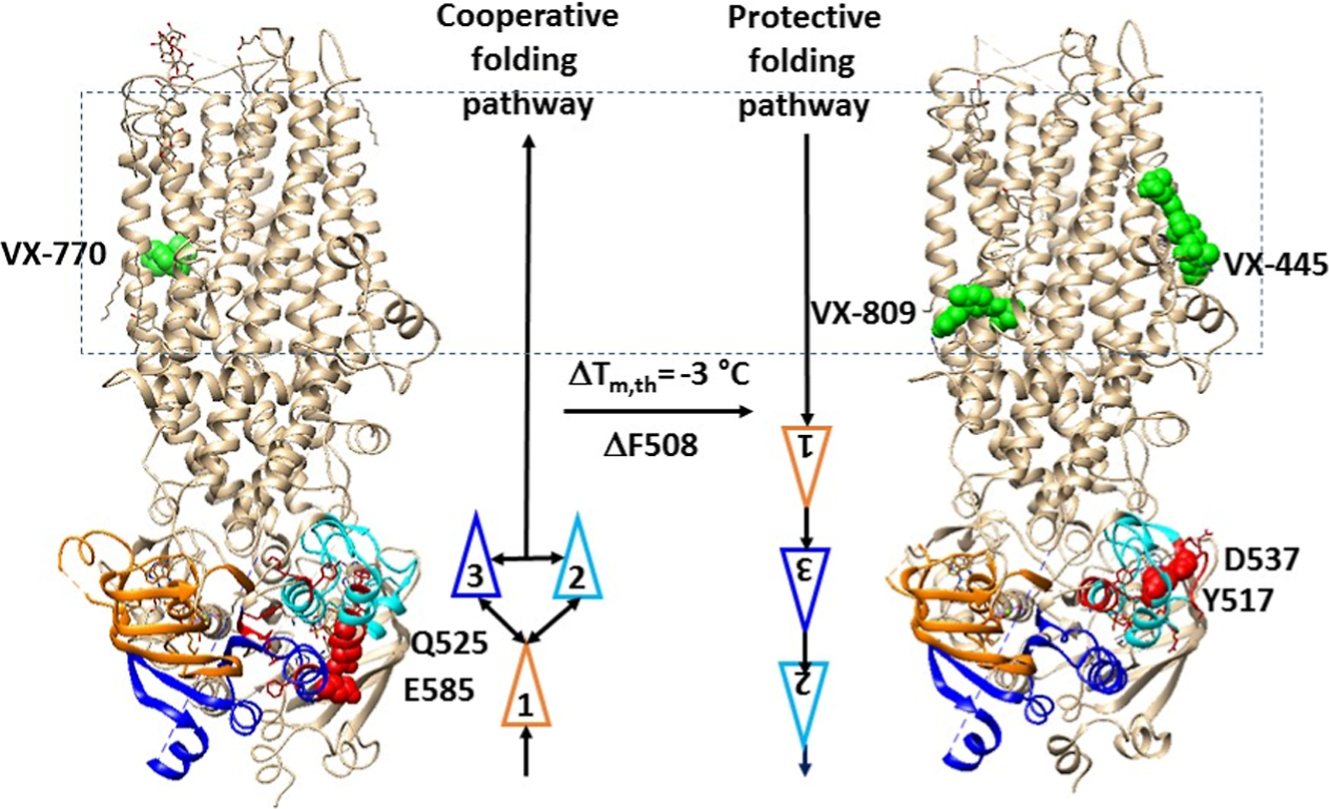
Cooperative and protective folding pathways of NBD1 in hCFTR in response to modulators. Cryo-EM structures of activated phosphorylated hCFTR/E1371Q with Mg/ATP/VX770 bound (PDB: 6O2P) and open phosphorylated hCFTR/ΔF508/E1371Q with Mg/ATP/VX809/VX445 bound (PDB: 8EIO) are used for the models. VX770, VX-445, and VX-809 are colored green. The N-terminal, α- and β-subdomains in NBD1 are colored orange, cyan, and blue, respectively. The biggest thermorings in NBD1 are shown in red. The residues responsible for the least-stable noncovalent interactions in NBD1 are shown in space fills.

### Protective Folding Pathway in Dimerized (F508del)­hNBD1 in Response to Folding Correctors

A previous study revealed that delaying α-subdomain compaction favors cotranslational folding of isolated hNBD1.^3,12^ This study further demonstrated that finalizing α-subdomain compaction also facilitates post-translational folding of dimerized NBD1 in full-length hCFTR with or without F508 in response to folding correctors. Following the binding of a single folding corrector VX-809 or VX-445, the weakest D529–R555 salt bridge was always present in the α-subdomain of dimerized or partially dimerized NBD1 (Figures 3a and 4a).^30^ Similarly, when two folding correctors VX-445 and VX-809 or VX-661 bind to (F508del)­hCFTR, the weakest Y517-D537 H-bond also appeared in the α-subdomain of dimerized NBD1 (Figure 1a).^30^ These results suggested that the formation of the weakest D529–R555 or Y517–D537 bridge was the last event in the post-translational folding pathway of dimerized NBD1 in the presence of folding correctors.

Notably, type I correctors have been reported to enhance the stability of (F508del)­hCFTR at the plasma membrane at 37 °C.^46–48^ However, VX-445 does not provide enough NBD1 stability for poorly responsive variants,^32^ consistent with the lower *T*_m,th_ of 39 °C of partially dimerized NBD1 in (F508del)­hCFTR/E1371Q with VX-445 bound.^30^ This study further indicated that the combination of VX-445 with VX809 is needed for the higher *T*_m,th_ of 47 °C to unfold dimerized NBD1 in (F508del)­hCFTR/E1371Q (Figure 1c,d, Table 1).^30^ Similarly, when Trikafta modulators bind to (F508del)­hCFTR, the *T*_m,th_ needed to unfold dimerized NBD1 also increases to 49 °C.^30^ Thus, the additional binding of the potentiator VX770 may account for why Trikafta significantly boosts the activity of (F508del)­hCFTR more than VX-445 and VX-809 at 37 °C.^48^

On the other hand, folding correctors lumacaftor/VX-809 and presumably tezacaftor/VX-661 have been shown to optimize CFTR folding during synthesis in cells.^34^ This suggests that the folding corrector may act as a chaperone, allosterically regulating the critical D529–R555 or Y517–D537 bridge to ensure proper cotranslational folding of NBD1. Therefore, the type-I folding correctors VX-809 and VX-661 not only stabilize TMD1 early in biogenesis^49^ but also initiate a protective pathway to enhance the efficiency of NBD1 folding from cotranslation to posttranslation. This ultimately corrects structural defects for proper (F508del)­hCFTR synthesis and function.

### D529–R555 Salt Bridge Primes Correct NBD1 Folding

A recent study identified three conserved thermoring anchors in isolated NBD1 monomer or dimer with or without F508 or 3S mutations.^29^ The first is the smallest Grid_0_ formed by the T465–Mg–Q493 and K464/T465/S466–ATP bridges in the N-terminal subdomain upon Mg/ATP binding. The second is the smaller Grid_2_ shaped by Q552–D529–R555 H-bonds in the α-subdomain. The third is the smaller Grid_3_ lined by the E583–K606 and F587–H609 bridges in the β-subdomain. Although these three smaller thermorings are also conserved in dimerized NBD1 of full-length hCFTR/E1371Q,^30^ the binding of modulators to TMD1 or TMD2 or their interface disrupted the D529–Q552 or E583–K606 H-bond in dimerized NBD1 of hCFTR/E1371Q (Figures 1a, 2a, 3a, 4a).^30^ More importantly, the binding of single folding corrector VX-661, VX-809, or VX-445 rendered the D529–R555 salt bridge the weakest in NBD1 no matter whether F508del is introduced or not (Figures 3a and 4a).^30^ Therefore, despite complex CFTR NBD1 folding kinetics, the highly conserved D529–R555 salt bridge is always required for normal NBD1 folding upon Mg/ATP binding. In support of this proposal, the D529F or R555 K mutation significantly improves NBD1 and full-length CFTR folding,^24^ possibly by restoring or enhancing kinetic coupling between α-helical subdomain and β-sheet core (Figure 2a), or finalizing the folding of α-helical subdomain (Figures 3a and 4a).^3,12,29,30^ On the contrary, as the nearby L558S mutation disrupts the coordinated compaction of α-helical and β-sheet core subdomains, which cannot be restored by the introduction of S492P and I539T (PT),^50^ this critical D529-R555 salt bridge may be kinetically and thermodynamically impaired. Therefore, no matter whether a cooperative or protective folding pathway is used during CFTR biosynthesis, the highly conserved D529–R555 salt bridge, together with the highly conserved Mg/ATP binding site, is always needed for correct NBD1 folding in response to complex environment perturbations. Further mutations and molecular dynamics simulations in this region are required to illuminate the protective role of the salt bridge in NBD1 folding and CFTR trafficking with or without the involvement of folding modulators.

## Conclusions

Tertiary noncovalent interactions such as H-bonds, π interactions, and salt bridges play a crucial role in protein folding and stability. However, the energy landscape of these interactions is not fully understood. Based on high-resolution 3D structures of NBD1 from hCFTR, tertiary noncovalent interaction networks were constrained as “thermorings” of various sizes from the smallest to the biggest to control the melting temperature of each noncovalent interaction. This allows for minimal energy requirements to regulate the timing of protein biosynthesis in different scenarios. Although chaperones are typically used to protect a subdomain from cotranslational misfolding in the ribosome, the weakest noncovalent link can also protect a subdomain from posttranslational misfolding. This is essential for proper protein function in response to chemical perturbations. These findings underscore the significance of an intrinsic thermodynamic protection strategy in the post-translational folding of proteins and the management of relevant inherited diseases.

## Supplementary Material



## Data Availability

All data generated or analyzed during this study are included in this published article and Supporting Information

## Notes

Thermoring structures ranging from small organic molecules to biomacromolecules; identified tertiary noncovalent interactions along the single peptide chain of NBD1 from L383 to L636 in hCFTR/E1371Q/ΔF508 with MgATP/VX445/VX809 bound at 4 °C (PDB ID, 8EIO); identified tertiary noncovalent interactions along the single peptide chain of NBD1 from E384 to Q637 in hCFTR/E1371Q with MgATP/VX770 bound at 4 °C (PDB ID, 6O2P); identified tertiary noncovalent interactions along the single peptide chain of NBD1 from E384 to Q637 in hCFTR/E1371Q with MgATP/VX661 bound at 4 °C (PDB ID, 7SV7); and identified tertiary noncovalent interactions along the single peptide chain of NBD1 from E384 to Q637 in hCFTR/E1371Q with MgATP/VX809 bound at 4 °C (PDB ID, 7SVD) (PDF)

## Abbreviations

ABC: ATP-binding cassette
CF: cystic fibrosis
cryo-EM: cryoelectron microscopy
CFTR: cystic fibrosis transmembrane conductance regulator
hCFTR: human CFTR
ICLi: intracellular loop *i* (*i* = 1,2,3,4)
MD: molecular dynamics
NBD1: nucleotide binding domain 1
NBD2: nucleotide binding domain 2
PKA: protein kinase A
R: regulatory
RE: regulatory extension
RI: regulatory insert
*T*_i_: systematic thermal instability
*T*_m,th_: melting temperature threshold
TMD1: transmembrane domain 1
TMD2: transmembrane domain 2
