# Supplementary material for: Thermodynamic Coupling between Folding Correctors and the First of Dimerized Nucleotide Binding Domains in CFTR
Source: ACS Bio Med Chem Au. 2025 Jul 30;5(4):593–601. doi: 10.1021/acsbiomedchemau.5c00014 (PMC12371502; doi:10.1021/acsbiomedchemau.5c00014)
Supplement: Supplementary file 1 [file bg5c00014_si_001.pdf]

Supporting Information for

Thermodynamic Coupling between Folding Correctors and the First of Dimerized Nucleotide Binding Domains in CFTR

Guangyu Wang[1,2]*

[1]Department of Physiology and Membrane Biology, University of California School of Medicine, Davis, California 95616, United States

[2]Department of Drug Research and Development, Institute of Biophysical Medico-chemistry, Reno, Nevada 89523, United States

*E-mail: gary.wang10@gmail.com

This Supporting Informational includes:

- Thermoring structures ranging from small organic molecules to biomacromolecules 1-4
- Identified tertiary noncovalent interactions along the single peptide chain of NBD1 from L383 to L636 in hCFTR/E1371Q/ΔF508 with MgATP/VX445/VX809 bound at 4 °C (PDB ID, 8EIO) 5
- Identified tertiary noncovalent interactions along the single peptide chain of NBD1 from E384 to Q637 in hCFTR/E1371Q with MgATP/VX770 bound at 4 °C (PDB ID, 6O2P) 6
- Identified tertiary noncovalent interactions along the single peptide chain of NBD1 from E384 to Q637 in hCFTR/E1371Q with MgATP/VX661 bound at 4 °C (PDB ID, 7SV7) 7
- Identified tertiary noncovalent interactions along the single peptide chain of NBD1 from E384 to Q637 in hCFTR/E1371Q with MgATP/VX809 bound at 4 °C (PDB ID, 7SVD) 8

Thermoring structures ranging from small organic molecules to biomacromolecules

In 1965, a concept of "thermal ring" was utilized for the stereo-selective thermosensitive electrocyclic reaction, based on Woodward–Hoffmann rules.[1] For instance, regarding the same *cis*- and *trans*-configuration, the temperature threshold is 170 °C for the thermal ring-opening of 3,4-dimethylcyclobutene but only 130 °C for the thermal ring closure of the 2,4,6-octatriene to 5,6-dimethyl-1,3-cyclohexadiene.[2-3] It is evident that a higher temperature is necessary for the opening of the smaller thermal ring.

From 2008 to 2013, it has been reported that an intramolecular RNA or DNA hairpin in a single polynucleotide chain can act as a thermal sensor. The melting temperature threshold ($T_{m,th}$) upon the decyclization of the loop is controlled not only by the number of H-bonds in the stem but also by the loop length.[4-6] Generally, in 150 mM NaCl, for a DNA hairpin with a 20 base long poly-A loop and two G-C base-pairs in the stem, the $T_{m,th}$ is about 34 °C. This value can be further increased by 10 °C with one additional G-C base-pair in the stem, or by 2 °C with one additional base in the loop.[6] However, the concept of a "thermal ring" has not been created to define such a thermosensitive RNA or DNA hairpin.

On the other hand, since 2001, a graph theory-based network analysis of noncovalent interactions has been employed to simulate thermal unfolding or thermostability engineering protein structures.[7-12] For example, in 2019, Residue Interaction Networks (RINs) with energy weighing were carried out at a secondary structure level to predict melting temperatures (T_m) of proteins. However, circular dichroism (CD) and differential scanning calorimetry (DSC) of the first nucleotide binding domain (NBD1) isolated from cystic fibrosis transmembrane conductance regulator (CFTR) showed that protein melting is initiated from the heat-induced unfolding in the tertiary structure, rather than the secondary structure.[13] This may account for why the performance of RINs only permits the distinction between [12]thermostable and mesostable proteins with an accuracy of 76%.

In 2011, rigid theory-based constraint network analysis (CNA) was used to analyze noncovalent interactions such as H-bonds or salt bridges in order to calculate the differences in phase-transition temperatures (T_p) between a mesophilic protein and its thermophilic counterpart. While the results were somewhat similar to the variances in experimental optimal growth temperatures,[9] this approach did not accurately predict the full range of activity temperatures for a protein, from its initial point to peak and then decline.

In 2023, a graph theory-based grid thermodynamic model was created to uncover the network basis for the temperature-dependent structure-function relationships of class I fructose bisphosphate (FBP) aldolase B and class II *E.coli* FBP aldolase.[14-16] Specifically, the noncovalent interaction networks along a polypeptide chain at the tertiary structural level can be constrained as topological "grids" of various sizes from the biggest to the smallest to regulate the weakest noncovalent interaction in the grids. Thus, when the minimal energy required to stabilize the weakest noncovalent interaction in the grid is also adjusted by the grid size, all the noncovalent interactions can be energetically ranked from the weakest to the strongest along the entire polypeptide chain. In this case, when the temperature-dependent structure-function relationships of class I and II FBP aldolases are closely related to the thermal unfolding of specific grids,[14-16] these grids become essentially thermo-sensitive. Consequently, when a grid-like network is utilized in the canonical topologies,[17] a concept of a "thermal ring" was initially introduced to define the specific "grids" in the constrained networks of tertiary noncovalent interactions that facilitate the protein folding.[16]

Given that the "thermal gradient ring" or "thermal ring" was also used to define temperature-dependent behaviors of transient receptor potential (TRP)-deficient mice by routing mice along the thermosensitive ring floor,18 a concept of "thermoring" or "thermo-ring" has been further created to predict a temperature-dependent conformational change or folding/unfolding that relates to thermal activation and inactivation of a protein.[19-24] In addition, as the ratio of the total grid sizes to the total non-covalent interactions along a specific polypeptide chain can be employed to evaluate proteins's compact conformational entropy or flexibility, the ratio is also defined as a systematic thermal instability (T_i) of protein.[14-16, 19-24] Finally, since the thermoring can absorb more heat as configurational heat capacity to minimize conformational entropy at elevated temperatures,[25] a structural temperature coefficient or sensitivity (Ω_{10}) of protein activity can also be predicted once the temperature-dependent thermoring structures are available.[19-22]

Taken together, while the concept of the "thermal ring" has been extensively used in organic chemistry, the concept of "thermoring" has just been introduced into biochemistry or biophysics. Further case studies are necessary to examine if this new concept can be expanded to predict the structural thermo-stability and the functional thermo-activity of other biological macromolecules, promoting rational and precise engineering design or disease management.

References

1. Hoffmann R. and Woodward R. B. Conservation of orbital symmetry. *Accounts of Chemical Research.* **1968,** 1, 17-22.
2. Marvell E. N., Caple G., Schatz B. Thermal valence isomerizations: stereochemistry of the 2,4,6-octatriene to 5,6-dimethyl-1,3-cyclohexadiene ring closure. *Tetrahedron Lett.* **1965**, 6, 385-389.
3. Rudolf E., Winter. K. The Preparation and Isomerization of cis- and trans- 3,4-dimethylcyclobutene. *Tetrahedron Letters,* **1965**, 6, 1207-1212.
4. Kuznetsov S.V., Ren C.C., Woodson S. A., Ansari A. Loop dependence of the stability and dynamics of nucleic acid hairpins. *Nucleic Acids Res.* **2008,** 36, 1098-1112.
5. Ke G., Wang C., Ge Y., Zheng N., Zhu Z., Yang C. J. L-DNA molecular beacon: a safe, stable, and accurate intracellular nano-thermometer for temperature sensing in living cells. *J Am Chem Soc.* **2012,** 134, 18908-18911.
6. Jonstrup A. T., Fredsøe J., Andersen A. H. DNA Hairpins as Temperature Switches, Thermometers and Ionic Detectors. *Sensors.* **2013,** 13, 5937-5944.
7. Jacobs D. J., Rader A. J., Kuhn L. A., Thorpe M. F. Protein flexibility predictions using graph theory. *Proteins* **2001,** 44,150–165.
8. Vishveshwara S. Brinda K. V., Kannan N. Protein structure: insights from graph theory. *J. Theor. Comput. Chem.,* **2002,** 1, 187–211.
9. Radestock S., Gohlke H. Protein rigidity and thermophilic adaptation. *Proteins.* **2011,** 79, 1089-1108.
10. Pfleger C., Rathi P. C., Klein D. L., Radestock S., Gohlke H. Constraint Network Analysis (CNA): a Python software package for efficiently linking biomacromolecular structure, flexibility, (thermo-)stability, and function. *J Chem Inf Model.* **2013,** 53,1007-1015.
11. Nutschel C., Fulton A., Zimmermann O., Schwaneberg U. Jaeger K. E., Gohlke H. Systematically scrutinizing the impact of substitution sites on thermostability and detergent tolerance for bacillus subtilis lipase A. *J Chem Inf Model.* **2020,** 60, 1568-1584.

12. Miotto M., Olimpieri P. P., Di Rienzo L., Ambrosetti F., Corsi P., Lepore R., Tartaglia G. G., Milanetti E. Insights on protein thermal stability: a graph representation of molecular interactions. *Bioinformatics.* **2019,** 35, 2569-2577.
13. Protasevich I, Yang Z, Wang C, Atwell S, Zhao X, Emtage S, Wetmore D, Hunt J. F., Brouillette C. G. Thermal unfolding studies show the disease causing F508del mutation in CFTR thermodynamically destabilizes nucleotide-binding domain 1. *Protein Sci.* **2010**, 19,1917-1931
14. Wang G. The network basis for the structural thermostability and the functional thermoactivity of aldolase B. *Molecules.* **2023**, 28(4), 1850.
15. Wang G. The network basis for the heat-adapted structural thermostability of bacterial class II fructose bisphosphate aldolase. *ACS Omega* **2023,** 8 (20), 17731−17739.
16. Wang G. Thermal ring-based heat switches in hyperthermophilic class II bacterial fructose aldolase. *ACS Omega.* **2023**, 8, 24624–24634.
17. Gosztolai A, Arnaudon A. Unfolding the multiscale structure of networks with dynamical Ollivier-Ricci curvature. *Nat Commun*. **2021,** 12, 4561.
18. Ujisawa T, Lei J, Kashio M, Tominaga M. Thermal gradient ring for analysis of temperature-dependent behaviors involving TRP channels in mice. *J Physiol Sci.* **2024**, 74(1), 9.
19. Wang G. Thermoring-Based Heat Activation Switches in the TRPV1 Biothermometer. *Int. J. Biol. Macromol.* **2023**, 248, 125915.
20. Wang G. Thermoring basis for the TRPV3 bio-thermometer. *Sci Rep.* **2023;** 13, 21594.
21. Wang G. Phosphatidylinositol-4,5-biphosphate (PIP_2)-Dependent Thermoring Basis for Cold-Sensing of the Transient Receptor Potential Melastatin-8 (TRPM8) Biothermometer. *Physchem.* **2024,** 4, 106–119.
22. Wang G. Thermo-ring basis for heat unfolding-induced inactivation in TRPV1. *Nat. Sci.* **2024**, 4, e20240008.
23. Wang G. ATP-dependent thermoring basis for the heat unfolding of the first nucleotide-binding domain isolated from human CFTR. *Nat Sci.* **2025,** 5(1-2), e70007.
24. Wang G. Trikafta rescues F508del-CFTR by tightening specific phosphorylation-dependent interdomain interactions. *Nat Sci.* **2025**, 5(3), e70009.
25. Karshikoff A., Nilsson L., Ladenstein R. Rigidity versus flexibility: the dilemma of understanding protein thermal stability. *FEBS J.* **2015,** 282, 3899–3917.

Table S1. Identified tertiary noncovalent interactions along the single peptide chain of NBD1 from L383 to L636 in hCFTR/E1371Q/ΔF508 with MgATP/VX445/VX809 bound at 4 °C (PDB ID, 8EIO)

Noncovalent interaction	Cut-off distance	Linked residues
Salt/metal bridge	3.2-4 Å	**T465/Q493/**D572-**Mg^{2+}**, **D529-R555**
H-bond	<3.9 Å donor-H-acceptor <60°	N396-N445, W401-S466, K442-S623, K447-Y627, L453-D614, **K464/T465/S466/Q493-ATP**, W496-R560, K503-Y512, **Y512-E514**-R518**, D513-Y563**, R516-Y563, **Y517-D537**, **E527/E528-S531, D529-Q552**, E535-N538, **A566-Y569**, D565-K598, **D579-T582**, K584-E588, E608-K611, T629-S631-N635
π−π interaction	2.65–6.5 Å	**W401-ATP**, **Y512-Y517**, **F575-H609-F587-F575**
cation−π interaction	<6.0 Å	
CH_3/CH−π interaction	2.65-3.01 Å	**F446**-L454, **W401-L475**, **M472-F490**, **P574-F575,** H620-E621, F626-L633
Lone pair−π interaction	3-3.7 Å	**Y569-M595, Y587-C692**

Note: Bold interactions were shared between hCFTR/E1371Q and hCFTR/E1371Q/ΔF508.

Table S2. Identified tertiary noncovalent interactions along the single peptide chain of NBD1 from E384 to Q637 in hCFTR/E1371Q with MgATP/VX770 bound at 4 °C (PDB ID, 6O2P)

Noncovalent interaction	Cut-off distance	Linked residues
Salt/metal bridge	3.2-4 Å	**T465/Q493/**D572-**Mg^{2+},** K503-D537, D529-R555
H-bond	<3.9 Å donor-H-acceptor <60°	E391-K447, T398-L441, K447-Y627, A462-G622, G463/**K464/T465/S466/Q493-ATP**, T465-D572, **W496-R560, Y512-E514**, **D513-Y563, Y517-D537**, Q525-E585, **E527/E528-S531,** K564-D565, K564/**A566-Y569, D579-T582**, **E583-K606**, T629-S631
π–π interaction	2.65–6.5 Å	F400-F409, **W401-ATP**, W496-F508, **Y512-Y517**, Y517-Y563, **F575-H609-F587-F575**
cation–π interaction	<6.0 Å	**K503-Y512,** R516-Y563
CH_3/CH–π interaction	2.65-3.01 Å	**M394-F446**, **W401-L475**, **M472-F490**, F508-R560, **Y517-I521**, F533-I539, **P574-F575,** F626-L633
Lone pair–π interaction	3-3.7 Å	**F587-C592**

Note: Bold interactions were shared between hCFTR/E1371Q and hCFTR/E1371Q/ΔF508.

Table S3. Identified tertiary noncovalent interactions along the single peptide chain of NBD1 from E384 to Q637 in hCFTR/E1371Q with MgATP/VX661 bound at 4 °C (PDB ID, 7SV7)

Noncovalent interaction	Cut-off distance	Linked residues
Salt/metal bridge	3.2-4 Å	E391-K447, **T465/Q493-Mg^{2+}**, K522-E527, E588-K612
H-bond	<3.9 Å donor-H-acceptor <60°	N396-D443-S624, K447-Y627, T460/G461/**K464/T465/S466/Q493-ATP**, A462-G622-K442, T465-D572, W496-R560, T501-E504, D511/**D513-Y563**, **Y512-E514**, **Y517-D537**, **E527/E528-S531, D529-R555**, K564/**A566-Y569**, D567-T599, **D579-T582**
π–π interaction	2.65–6.5 Å	**W401-ATP**, W496-F508, **Y512-Y517**, **F575-H609-F587-F575**
cation–π interaction	<6.0 Å	**K503-Y512**
CH_3/CH–π interaction	2.65-3.01 Å	**W401-L475**, **M472-F490**, F508-R560, F533-I539, **P574-F575**
Lone pair–π interaction	3-3.7 Å	Y515-S519, **Y569-M595**, **F587-C592**

Note: Bold interactions were shared between hCFTR/E1371Q and hCFTR/E1371Q/ΔF508.

Table S4. Identified tertiary noncovalent interactions along the single peptide chain of NBD1 from E384 to Q637 in hCFTR/E1371Q with MgATP/VX809 bound at 4 °C (PDB ID, 7SVD)

**Noncovalent interaction**	**Cut-off distance**	**Linked residues**
Salt/metal bridge	3.2-4 Å	**T465/Q493-Mg^{2+}**
H-bond	<3.9 Å donor-H-acceptor <60°	K442-S623, K447-Y627, L453-F614, T460/**K464/T465/S466/Q493-ATP**, T465-D572, W496-R560, T501-E504, K503-Y512, **Y512-E514**, **D513-Y563**, **E527/E528-S531, D529-R555**, **A566-Y569**, S549-Q552, **D579-T582**, **E583-H609**, E608-K611
π–π interaction	2.65–6.5 Å	**W401-ATP**, W496-F508, **Y512-Y517**-Y563, **F575-H609-F587-F575**
cation–π interaction	<6.0 Å	
CH_3/CH–π interaction	2.65-3.01 Å	**W401-L475**, **M472-F490**, F508-R560, **Y517-I521**, **P574-F575,** F626-L633
Lone pair–π interaction	3-3.7 Å	**Y569-M595**, **F587-C592**

Note: Bold interactions were shared between hCFTR/E1371Q and hCFTR/E1371Q/ΔF508.
